# Comparison of the Cardiovascular Effects of Extreme Psychological and Physical Stress Tests in Male Soccer Players

**DOI:** 10.3390/ijerph19020715

**Published:** 2022-01-09

**Authors:** Ákos Móra, Zsolt Komka, József Végh, István Farkas, Gyöngyi Szilágyi Kocsisné, Edit Bosnyák, Márta Szmodis, Roland Ligetvári, Éva Csöndör, Gábor Almási, András Oláh, Han C. G. Kemper, Miklós Tóth, Pongrác Ács

**Affiliations:** 1 Faculty of Health Sciences, University of Pécs, 7621 Pécs, Hungary; roland.ligetvari@gmail.com (R.L.); eva.csondor@gmail.com (É.C.); andras.olah@etk.pte.hu (A.O.); tothmik1@hotmail.com (M.T.); pongrac.acs@etk.pte.hu (P.Á.); 2 Department of Health Sciences and Sport Medicine, University of Physical Education, 1123 Budapest, Hungary; komkazsolt@gmail.com (Z.K.); bosnyaked@gmail.com (E.B.); szmodis.marta@tf.hu (M.S.); alm.gabor@gmail.com (G.A.); 3 International Training Centre, 1126 Budapest, Hungary; vege@elitkft.hu (J.V.); farkas.istvan@nokitc.hu (I.F.); 4 I-QRS International Ltd., 1165 Budapest, Hungary; szilagyi@iqrs.hu; 5 Medical Centers, Amsterdam University, 1081 Amsterdam, The Netherlands; hancgkemper@upcmail.nl; 6 Department of Laboratory Medicine, Semmelweis University, 1089 Budapest, Hungary; 7 Szentágothai Research Centre, University of Pécs, 7624 Pécs, Hungary

**Keywords:** heart rate variability, blood pressure, physical stress, psychological stress

## Abstract

Background: The purpose of our study was to compare the physiological effects of extreme physical and psychological stress tests in male soccer players, since these two types of stress apply to athletes with high performance requirements. Methods: A total of 63 healthy male soccer players participated in this study, all of whom underwent both of the tests. A physical stress test was carried out in an exercise physiology laboratory, where subjects completed an incremental treadmill running test to full exhaustion, and a psychological test was performed in a military tactical room, where subjects met a street offence situation. Heart rate variability (HRV) and blood pressure (BP) were recorded directly before, immediately after, and 30 min after the stress tests. Results: The majority of HRV indices changed significantly in both stress protocols. Inverse, significant changes (positive for the physical test, negative for the psychological test, *p* < 0.001) were found when comparing the alterations of HRV indices between the tests. Significant differences were found in the changes in systolic (*p* = 0.003) and diastolic (*p* < 0.001) BP between the test protocols, and also between the baseline and post-test measurements (*p* < 0.001). Conclusion: Both HRV and BP are sensitive physiological parameters to measure the impact of extreme physical and/or psychological stress

## 1. Introduction

Stress is the human body’s process of coping with acute or prolonged stressors in general or specific situations. Different stressors may trigger various responses that lead to changes in the physiological and psychological state of the human body [1]. Since stress is managed by the autonomic nervous system (ANS), in the field of sports this coping process can be motivating (enhances sport performance) or demotivating (reduces sport performance), depending on the relevant earlier experiences. Stress affects athletes both physically and mentally at the same time, and their combined impact can be measured using heart rate variability (HRV) monitoring. HRV is a physiological phenomenon that analyzes the variation in time between consecutive heart cycles; it is a very sensitive indicator of ANS activity, and also a reliable reflection of the physiological factors that control the normal rhythm of heartbeats [2]. HRV can be easily measured using laboratory equipment (e.g., electrocardiogram (ECG)), or even wearable devices (several heart rate monitors), both providing valid data in case of a normal sinus rhythm. This method is non-invasive and has a good reproducibility if used under standardized conditions.

Modern life is becoming increasingly stressful, so understanding how to deal with environmental stressors is essential in order to create effective coping methods [3]. The physiological changes in the human body induced by a physical or psychological stress differ in many ways. There are both acute and long-lasting responses in the neuroendocrine, cardiovascular, and respiratory systems, and also in the muscles [4]. Out of many different known stressors, the psychosocial stressors have the most profound effect on the cardiovascular system [5]. The human body’s immediate reaction to a stressful situation is the fight-or-flight response (increased level of the hormone epinephrine, elevation of heart rate, blood pressure, rate of breathing, etc.), whereby the sympathetic nervous system (SNS) prepares the body for different reactions [6,7]. The changes in HRV caused by physical and psychological stress are not a deeply discussed area of the effects of stress, although they do represent a sensitive physiological variable. The responses of the cardiovascular system to different stress types are distinct, and it remains unclear how HRV indices change in response to these extreme interventions.

Many researchers have analyzed the relationship between psychological stress and HRV, mostly using the validated job stress questionnaire, along with basic cardiac measurements (e.g., ECG). The most frequently reported factor is low parasympathetic activity and, consequently, increased sympathetic activity, resulting in a decrease in the high-frequency (HF) band and an increase in the low-frequency (LF) band [8]. These findings have been confirmed by numerous studies [9,10,11], although the LF and the LF/HF ratio parameters are still controversial—sympathovagal balance cannot be accurately measured via the ratio of LF to HF. In the field of sport science, psychological stress can also be measured as pre-competitive anxiety, which has also been examined through HRV measurements in numerous studies [12,13,14]. In addition to HRV, the most frequently reported objective evaluation of stress levels is the measurement of the hormone cortisol. According to the literature, the level of cortisol increases after physical stress [15].

HRV has also been analyzed in special situations. Urban combat is a situation that creates high-level psychological stress, regardless of the setting being real or simulated. Despite the low physical intensity (slow movements), psychological stress can increase the heart rate (HR) [16]—a finding that was confirmed by Tornero-Aguilera et al. [17], and also by Kellerová [18]. A lower vagal HRV component was associated with pre and post-combat development of post-traumatic stress disorder (PTSD) symptoms. Many studies have shown that patients with PTSD have lower HRV indices than healthy adults [19,20,21,22]. 

Mancia and Grassi [23] state that it is possible that the increased sympathetic drive interacts with blood pressure (BP). Lee et al. [24] found that HRV indices were associated with an increase in systolic blood pressure. Systolic BP increases in a curvilinear manner as the load of treadmill running or cycling exercise increases; after the exercise, it takes 10–20 min to return to resting levels. Unlike systolic blood pressure, diastolic blood pressure remains constant, or even decreases by a few mmHg [25,26,27]. 

The aim of our study was to analyze and compare the cardiovascular effects of an extreme psychological and physical stress test, and to define possible relationships between the changes in BP and several HRV indices during any of the stress protocols. 

## 2. Materials and Methods

### 2.1. Participants and Design

A cross-sectional design was employed for this study. The population was a convenient sample of healthy, male, second-division soccer players (*n* = 63; age: 25.14 ± 5.81 y). Participants were intentionally chosen from team sports, since they also experience several interpersonal issues that create extra stress beyond the pressure of competition. Subjects underwent an extreme physical and psychological stress test. It was necessary to choose athletes, because the length of the different tests was planned to be roughly analogous, so that the physiological changes were more comparable. We created two groups based on subjects’ mean relative VO_2_peak (52.14 mL/kg/min; above average *n* = 29, below average *n* = 34), and also four groups based on the 25th (48.3 mL/kg/min), 50th (53.1 mL/kg/min) and 75th (57.9 mL/kg/min) percentiles of the relative VO_2_peak of the participants (Group 1: below the 25th percentile, *n* = 16; Group 2: between the 25th and 50th percentiles, *n* = 16; Group 3: between the 50th and 75th percentiles, *n* = 16; Group 4: above the 75th percentile, *n* = 15). Grouping was also performed based on the findings of Perna et al. [28] for the psychological stress protocol. Subjects who increased both their rMSSD (root mean square of successive differences) and HF values between the pre- and post-test measurements were assigned to group A (*n* = 32); the rest of the subjects were assigned to group B (*n* = 31).

### 2.2. Ethics

The subjects were treated in accordance with the principles of the Declaration of Helsinki. The Hungarian Research Ethics Committee approved the study protocol. Each individual was informed of the benefits and risks of the investigation prior to signing an informed consent document. Participants were informed that their participation was voluntary, and were given the option to terminate the test and their participation at any time during the test protocols.

### 2.3. Testing Protocols

The physical stress test was carried out in an exercise physiology laboratory, where participants performed a maximal incremental treadmill running test with a modified Bruce protocol (2 min of warm-up at 8 km/h speed, which was increased to 10 km/h and then remained constant. Elevation was 0% in the first 3 min, and was increased 1.5% after each minute). A PowerCube gas analyzer unit supplied by Ganshorn (Niederlauer, Germany) was used in order to measure peak VO_2_ values (VO_2_peak, highest 10-s average); the gas analyzer was calibrated before each measurement. Tests were terminated if a subject achieved maximal oxygen uptake criteria and was unable to continue (volitional fatigue). The basic criteria used to evaluate relative VO_2_peak were reaching the plateau in oxygen uptake, respiratory exchange ratio higher than 1.1, and heart rate equal to or higher than 90% of the age-predicted HRmax [29].

The extreme psychological stress test was carried out couple of days later. The psychological stress protocol was designed and published by a clinical psychologist [30,31]. A less complex, modified version of the protocol was used, and was implemented in a special military tactical room. Subjects received protective equipment and a dummy gun, along with usage instructions, 20 min before entering the room. The subjects had to enter the room, where a street view was installed, and had to walk through the room. Participants were told that an unexpected situation might take place during the test (e.g., people showing up), in which case subjects had to assess whether or not the situation represented a possible threat. After a while, a specialist entered suddenly from a different door, and fired his gun twice towards the subject, targeting beside the subject with dummy bullets in order to avoid injury, but creating the required stress effect. Other stress-enhancing factors included the appearance of a homeless-looking person, and the appearance of the shooter as a journalist in the same clothing. After a while, a whistle was blown, at which point participants had to put their guns down. The psychological stress test was carried out under the control of a well-trained military psychologist, because the maintenance of considerable sympathetic modulation for a long time may cause reductions in memory operability, special abilities, performance, or safety; this may lead to PTSD, which alters autonomic modulation, sleep quality, and resting heart rate, and can make the subjects feel unsafe. 

### 2.4. Data Dollection 

HRV data were recorded using a 12-lead ECG directly before (pre) and 30 min after the test (R30), whereas BP was measured before (pre), immediately after (post), and 30 min after the test (R30). The measurement of HRV was not carried out immediately after the test, because it was not possible to perform it in a standardized manner (supine position). The pre-test HRV and blood pressure measurements were used as the baseline parameters. Frequency- and time-domain HRV parameters were measured in a supine position, defined by 265 heart cycles measured by the 12-lead ECG, excluding the ectopic beats. Removal of artifacts was executed both by using the artifact correction option of Kubios HRV Standard software (Kubios Oy, Finland), and by manually selecting the ectopic-free RR intervals from the raw ECG data. Sampling frequency was 500 Hz. Frequency-domain analysis was performed using a fast Fourier transform. Subjects were asked to breathe normally during the measurement; breathing was not paced. For comparison of the physical and the psychological tests, we took into account the changes between before and after the tests (Δ values). Measured HRV indices were as follows: NNmax (longest difference in time of normal-to-normal beats, in ms), NNmin (shortest difference in time of normal-to-normal beats, in ms), NNmean (mean difference in time of normal-to-normal beats, in ms), SDNN (standard deviation of normal-to-normal heart cycle in time), pNN50 (ratio of consecutive RR interval pairs with at least 50 ms difference in time within the whole sample), rMSSD (root mean square of successive differences), TP (total power), VLF (very low frequency), LF (low frequency), HF (high frequency), and LF/HF ratio. Before the tests, blood pressure was measured in a sitting position; measurement was executed three times. The means of both the systolic and the diastolic values were used in the research. 

### 2.5. Statistical Analysis

Statistical analysis was carried out using SPSS 22 software (IBM, Chicago, IL, USA). To analyze differences between pre- and post-test data, and between the trends of physiological and psychological stress, a dependent *t*-test was performed. After grouping the participants based on their relative VO_2_peak value, we performed one-way ANOVA to examine the differences between the groups. Pearson’s correlation test was also performed in order to examine the linear correlation between the HRV parameters. All variables were assessed by Shapiro–Wilk test for normality of distribution. Calculation of effect size was also carried out (Cohen’s d and effect size r). A *p*-value < 0.05 was considered to indicate statistical significance (*: *p* < 0.05; **: *p* < 0.01; ***: *p* < 0.001). 

## 3. Results

All of the HRV parameters of the physical stress test increased significantly after the test, compared with the resting data. The difference was also significant for the majority of parameters of the psychological stress protocol—most values decreased in this test (Table 1). The NNmax, NNmean, pNN50, rMSSD, HF, and LF/HF ratio changed significantly in both the physical and psychological stress protocols.

**Figure 1:**
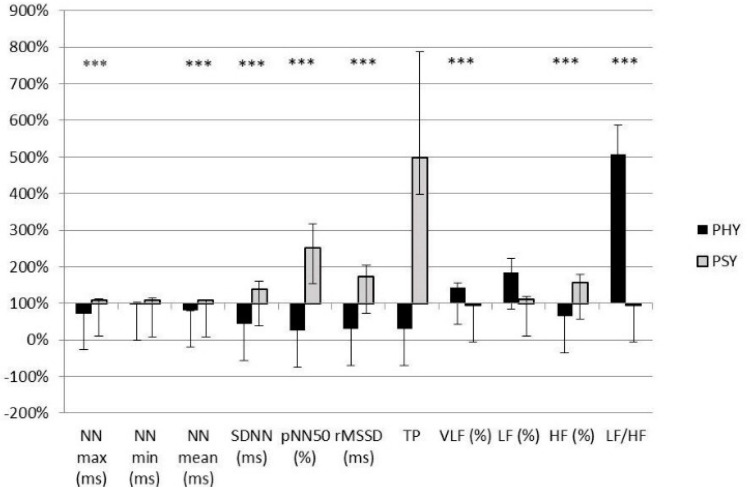
The different trends of HRV parameters between the physical (PHY) and psychological (PSY) stress tests (mean ± standard error of mean; ***: *p* < 0.001). The measurement of changes is expressed in percentages on the vertical axis. Abbreviations: see Table 1.

Comparison of the differences in the pre-test and R30 stress HRV measurements between the physical and psychological stress tests can be seen in Figure 1. Pre-test data are considered to be 100%, represented on the X-axis. The measurement of changes is expressed in percentages for both test protocols. Except for LF, all HRV components changed inversely. The differences in change of NNmax (ms), NNmean (ms), SDNN (ms), pNN50 (%), rMSSD (ms), VLF (%), HF (%), and LF/HF ratio between the stress protocols reached a high significance (*p* < 0.001). 

After grouping participants based on their average relative VO_2_peak, we found significant differences in the changes in LF/HF ratio for the physical stress test, and in the changes in the VLF component for the psychological stress test, between the groups (Table 2.). After creating four groups based on the percentiles of relative VO_2_peak, one-way ANOVA revealed significant differences between the four groups in the change in the VLF component for the psychological stress test (*p* = 0.033).

Pearson’s correlation test showed strong relationships between the delta values of many HRV indices; some of those relationships were found for both stress protocols. These correlations are given in Table 3. 

After performing the grouping based on the findings of Perna et al. (2019), we found significant differences in NNmax (*p* < 0.001), NNmean (*p* = 0.002), pNN50 (*p* = 0.048), VLF (*p* = 0.001), LF/HF ratio (*p* < 0.001), and VO_2_max (*p* = 0.041) between the groups. 

**Figure 2:**
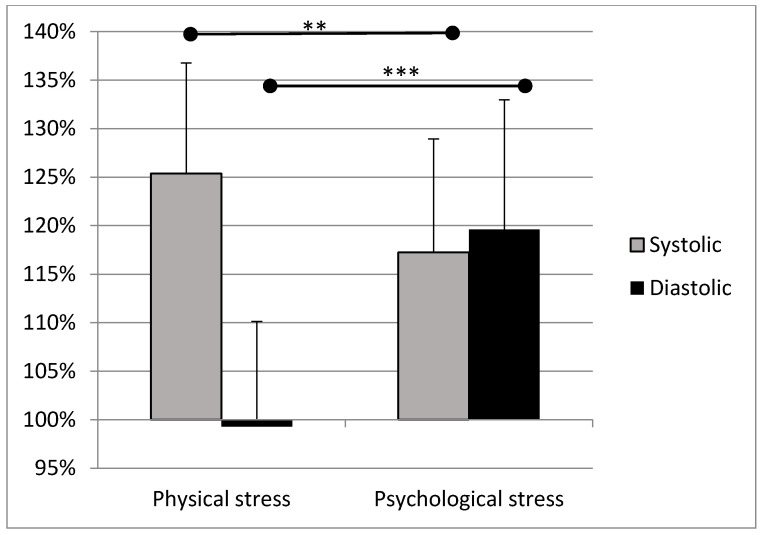
Average changes in blood pressure values between pre- and post-test. Pre-test data are considered to be 100%, represented by the X-axis. The measurement of change is expressed in percentages (vertical axis) for both test protocols. The differences in change in both systolic and diastolic BP results differed significantly between the test protocols (mean ± standard deviation; **: *p* < 0.01; ***: *p* < 0.001).

A significant difference was found in the systolic blood pressure between pre- and post-test measurements (34.4 ± 15.3 mmHg, *p* < 0.001) in the physical stress protocol. In the psychological stress test, both the systolic and the diastolic blood pressure values increased significantly (22.4 ± 14.6 mmHg, *p* < 0.001; and 14.1 ± 8.9 mmHg, *p* < 0.001, respectively) between the pre and the post-test measurements. We also analyzed the differences in changes in systolic and diastolic values between the pre- and post-test measurements. Significant differences were found in the changes in systolic (*p* = 0.003) and diastolic (*p* < 0.001) blood pressure between the test protocols (Figure 2). R30 blood pressure returned to the resting values. We could not detect any correlation between the changes in HRV parameters and blood pressure values.

## 4. Discussion

The purpose of our study was to compare the physiological effects of extreme physical and psychological stress tests in male soccer players, since these two types of stress apply to athletes with high performance requirements.

Strong correlations of certain HRV indices were predictable because of the correlations of their mathematical roots (e.g. SDNN - TP, or ΔSDNN - ΔTP). The SDNN parameter is the standard deviation of the N-N intervals—namely, the square root of variance—whereas TP is the total power of spectral analysis, which is mathematically equal to the variance. This relationship might explain why all HRV components that have correlation with ΔSDNN also showed a strong relationship with ΔTP. The European Heart Journal’s heart rate variability task force [32] also found a strong correlation between rMSSD and pNN50—a relationship that was confirmed by this study for both the physical (r = 0.69, *p* < 0.001) and psychological (r = 0.958, *p* < 0.001) stress tests. The aforementioned task force also revealed the approximate correspondence of HF with pNN50 and rMSSD in 24-hour recordings. Despite the short-term recording used, we found moderate correlation between these HRV indices for the physical stress protocol; however, no correlation was found for the psychological stress test. We could not confirm the findings of Hedelin et al. [33]—in our sample, higher relative VO_2_peak did not correlate with higher HF component. The appearance of this correlation was expected in our sample as well, because HF is an indicator of parasympathetic activity. Higher endurance performance (i.e., a higher VO_2_peak value) leads to a higher resting parasympathetic tone; however, our sample group was homogeneous in terms of VO_2_peak value. 

The vast majority of studies analyzing chronic stress [9,10,11] have reported a decrease in RR interval and significantly lower pNN50 and HF results in situations of psychological stress. According to Perna et al. [28], subjects with higher HF and rMSSD indices are able to deploy more neuropsychological resources that help them to overcome stressful situations. In our study subjects, who increased both their HF and rMSSD values between the pre-test and R30 measurements for the psychological stress test had decreased LF/HF ratios (0.68 ± 0.39) compared to the rest of the subjects (1.22 ± 0.63), i.e., the group that was supposed to be better at coping with psychological stress reached parasympathetic dominance. Since the effects of acute and prolonged stress are usually different, this finding conflicts with the aforementioned results. The decrease in RR interval and the lower pNN50 and HF components described by studies analyzing chronic stress indicate sympathetic superiority.

Clemente-Suárez and Robles-Perez [16] found that, in an urban combat situation, participants’ sympathetic dominance was increased, as could be seen from the decreasing mean RR and SDNN, showing the activation of the fight-or-flight defense system. Our study cannot confirm these findings, as we measured increasing NNmean and SDNN parameters; the opposing results may be explained by the differences in study design. The main difference is the sampling method, because the aforementioned author measured experienced soldiers, whereas our subjects were regular second-division soccer players, who experienced additional mental stress even before the protocol started. The timing and the device used for HRV measurement may also explain the different results, because the former authors obtained RR recording data before and immediately after the test, using wearable devices; their study design was specialized for soldiers, and subjects had to solve a complex mental task during the stress protocol, whereas in our study we demanded only an easy task (walking through the room) in order to avoid generating long-lasting psychological symptoms for our subjects. Based on our results of the psychological stress test, we cannot confirm the findings of studies analyzing pre-competitive anxiety [12,13] among cyclists, where an increase was found in sympathetic nervous system activity (decreased SDNN and rMSSD). Similarly to the above-mentioned study concerning an urban combat situation carried out with soldiers, athletes also increased their sympathetic dominance, unlike our participants during the psychological stress protocol. Presumably, our subjects were faced with a situation that they had never experienced before, whereas participants of the aforementioned studies were highly trained in the situations that the research protocol created. According to Thayer et al. [4], HRV reflects the flexibility of the cardiovascular system for coping with sudden stressors, meaning that HRV might be the index of the human body’s global capacity to regulate psychophysiological responses in an adaptive manner.

Our data suggest that experiencing an unknown, dangerous situation for the first time has a higher effect on HRV than any other factor. Previously mentioned studies examined stress levels using questionnaires, and during conditions participants encountered regularly. Our study subjects experienced extreme psychological stress in a military tactical room, leading to significant changes in the majority of HRV parameters. The different methods of measurement—in particular, the different levels of psychological stress reached in the aforementioned studies—explain the different results we obtained. An unknown situation in itself causes real stress in the human body, especially if there are some stress-enhancing circumstances generated, as in the case of our study (e.g., the paintball gun given to subjects). The lack of previous experience might also explain why our results contradict the findings of Perna et al. [28]. Adaptation to a situation influences the human body’s reaction; getting more experience in a certain situation changes the physiological response from the values of our study towards the values found in the previously mentioned references.

The changes in heart rate variability and blood pressure are two of the many cardiovascular responses of the human body to acute stress. The presence of a cardiovascular response does not require extreme stress [18]. The results of blood pressure data concur with the findings of Holland et al. [25], Instebø et al. [26], and Schultz and Sharman [27], since for the physical stress test only the systolic blood pressure increased, whereas for the psychological stress test both the systolic and diastolic values increased significantly. Lee at al. [24] found that all HRV parameters were associated with the changes in systolic blood pressure. We did not find any correlation between blood pressure and HRV values for either the physical or the psychological stress tests—neither among resting nor delta values. Wang et al. [34] analyzed the effect of a mental arithmetic task on blood pressure. The elevation of blood pressure that regularly occurs as a response to stress is most likely due to the increased sympathetic–adrenal–medullary activities; along with this, there is always an increased state of anxiety and tension. The observed changes in systolic and diastolic blood pressure between the pre- and post-test measurements are considered normal, and were expected to change accordingly. During physical stress, the aim of blood flow redistribution is to serve the increasing demands of working muscles. Higher flow velocity is necessary in order to increase cardiac output, which requires higher systolic blood pressure. Peripheral vascular resistance decreases because the capillary bed dilates to help the circulation redistribution, which results in decreasing diastolic blood pressure. Under psychological stress, adrenaline and noradrenaline cause vasoconstriction through the α1-adrenergic receptor, which increases both the systolic and diastolic blood pressure. Despite the different methods of psychological stress employed, our results confirm the findings of Wang et al. [34].

Although the analysis and the interpretation of HRV results are still controversial in science, our data show distinct trends. In our study, HRV parameters changed contrariwise between the test protocols, which can be explained by the presence or absence of the necessary experience in a given situation, based on the findings of international literature. This must be considered in a sporting environment, when athletes face the final of a major international tournament for the first time in their career, because their autonomic nervous system manages the stress differently. The most recent long-term stressor of athlete preparation was the consequences of the COVID-19 pandemic, which have been analyzed by several studies [35]. Further research is necessary in order to find other markers that signify the different regulation of the human body.

## 5. Strengths and Limitations

This study has both strengths and limitations. It is unique in terms of comparing the acute cardiovascular effects of extreme psychological and physical stress. The sample for the intervention contained only male soccer players. Analyzing HRV data as an objective and individually different response to acute psychological stress is a new aspect of observation of an athlete’s performance. Unfortunately, it was not possible to measure HRV immediately after the stress test; this third measurement could raise the level of novelty of the manuscript. The psychological stress protocol is not a commonly used method among soccer players, and was based on the original military testing protocol, although the subjects’ lack of experience in this kind of situation helped to create a more objective environment for the study. The comparison of the acute and prolonged effects is not accurate; as such, the defined consequences must be treated carefully. The findings of this research can be used in training situations. The number of participants was high enough to reveal the differences between the two types of stress, while the sample was homogeneous. Our results cannot be treated as general findings, due to the limitations of sampling (e.g., gender, age, sport).

## 6. Conclusions

In our study of soccer players, we compared the effects of physical and psychological stress tests on HRV and BP, measured before and after the tests. The results showed that both HRV and BP are sensitive physiological parameters to measure the impact of extreme physical or psychological stress. The increased or decreased HRV values remained unchanged for a longer period of time, whereas BP returned to the resting values earlier. In addition to the physical factors, many psychological impacts may affect athletes during competitions. The results suggest that psychological preparation is invaluable to athletes, in case they encounter an unknown, unexpected situation, because their ANS process the unknown stimuli differently. Usually, psychological preparation is implemented by sport psychologists; however, their methods should not be limited to theoretical preparation—athletes should be trained with practical psychological methods as well.

## Figures and Tables

**Table 1:** Dependent *t*-test results of comparison of pre- and post-test HRV parameters (Bold *p*-values indicate significant differences).

Test Protocol	HRV Parameter	Pre- vs. Post-Test Differences					
		Mean ± Standard Deviation	95% Confidence Interval of the Difference		Cohen’s d	Effect Size r	*p*
			Lower	Upper			
Physical test	NNmax	320.51 ± 180.19	275.13	365.89	1.85	0.68	**<0.001**
	NNmin	44.86 ± 144.19	8.54	81.17	0.38	0.19	**0.016**
	NNmean	185.22 ± 96.22	160.99	209.45	1.63	0.63	**<0.001**
	SDNN	50.41 ± 29.32	43.03	57.80	1.99	0.71	**<0.001**
	pNN50	26.11 ± 16.85	21.87	30.35	2.04	0.71	**<0.001**
	rMSSD	47.17 ± 30.90	39.39	54.96	1.94	0.69	**<0.001**
	TP	331,051 ± 3221.81	2499.11	4121.91	1.39	0.57	**<0.001**
	VLF	−6.10 ± 23.46	−12.00	−0.19	−0.34	−0.17	**0.043**
	LF	−8.11 ± 19.84	−13.11	−3.11	−0.54	−0.26	**0.002**
	HF	13.14 ± 14.25	9.56	16.73	1.20	0.51	**<0.001**
	LF/HF	−4.63 ± 5.60	−6.04	−3.22	−1.14	−0.49	<0.001
Psychological test	NNmax	−90.60 ± 198.53	−140.60	−40.60	−0.41	−0.19	**<0.001**
	NNmin	−38.79 ± 168.84	−81.32	3.73	−0.29	−0.15	0.073
	NNmean	−66.41 ± 84.58	−87.72	−45.11	−0.43	−0.21	**<0.001**
	SDNN	−7.46 ± 46.99	−19.29	4.37	−0.19	−0.09	0.212
	pNN50	−6.46 ± 13.59	−9.88	−3.04	−0.33	−0.16	**<0.001**
	rMSSD	−17.83 ± 47.31	−29.74	−5.91	−0.42	−0.21	**0.004**
	TP	−94.97 ± 6212.51	−1659.57	1469.63	−0.02	−0.01	0.904
	VLF	6.51 ± 20.72	1.29	11.73	0.41	0.20	**0.015**
	LF	−0.52 ± 13.95	−4.04	2.99	−0.04	−0.02	0.767
	HF	−5.59 ± 14.24	−9.17	−2.00	−0.39	−0.19	**0.003**
	LF/HF	0.40 ± 1.15	0.11	0.69	0.27	0.13	**0.007**

Abbreviations—NNmax: longest normal heart cycle during the measured period (ms); NNmin: shortest normal heart cycle during the measured period (ms); NNmean: average length of normal heart cycles during the measured period (ms); SDNN: standard deviation of normal heart cycles during the measured period (ms); pNN50: ratio of consecutive NN interval pairs with a greater difference than 50 ms (%); rMSSD: root mean square of successive differences (ms); TP: total power (ms^2^); VLF: very-low-frequency band; LF: low-frequency-band; HF: high-frequency-band; LF/HF: ratio of low- and high-frequency bands.

**Table 2:** Comparison of the changes in LF/HF ratio for the physical test and the changes in the VLF component for the psychological test between the two groups, based on the mean relative VO_2_peak (abbreviations: see Table 1; Bold *p*-values indicate significant differences).

Stress Test Protocol and HRV Parameter	Grouping Based on Mean Relative VO_2_peak	Mean ± SD	*p*	Mean Difference	Cohen’s d	Effect Size r	95% Confidence Interval of the Difference	
							Lower	Upper
Physical stress LF/HF Δ	Under	3.33 ± 3.19	**0.037**	−3.25	−0.55	−0.27	−6.29	−0.21
	Above	6.58 ± 7.64						
Psychological stress VLF Δ	Under	0.79 ± 0.38	**0.021**	−0.29	−0.63	−0.30	−0.54	−0.046
	Above	1.09 ± 0.56						

**Table 3:**
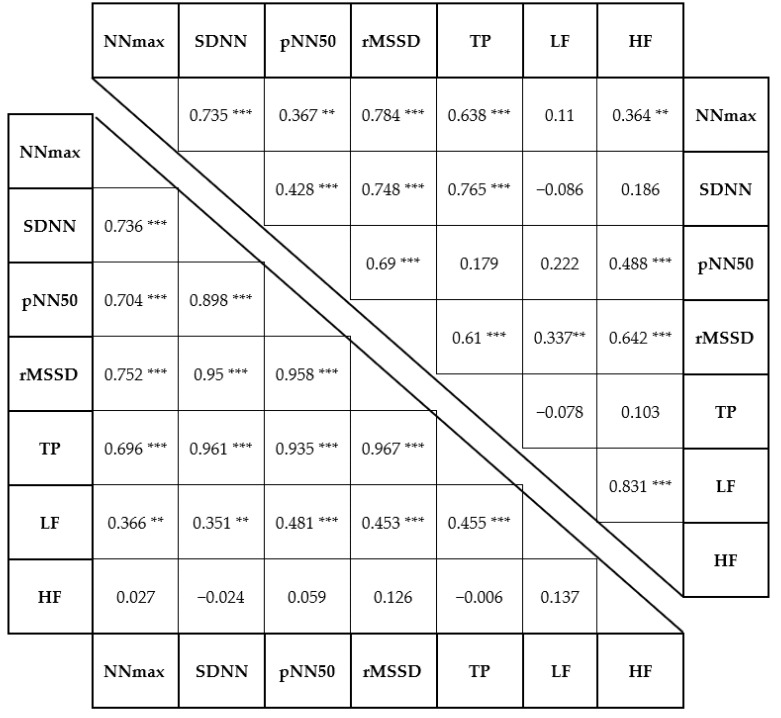
Correlations between the delta values of the HRV indicies. The results of the physical stress test can be seen in the upper right part of the table, whereas the buttom left part contents the results of the psychological stress protocol. Statistical significance is indicated by stars (** *p* < 0.01, *** *p* < 0.001). Abbreviations: see Table 1.

## Data Availability

The data presented in this study are available on request from the corresponding author. Some variables are restricted to preserve the anonymity of study participants.

## Abbreviations

ANS	Autonomic nervous system
BP	Blood pressure
ECG	Electrocardiogram
HF	High-frequency band
HRV	Heart rate variability
LF	Low-frequency band
LF/HF	Ratio of low- and high-frequency bands
NNmax	Longest normal heart cycle during the measured period
NNmean	Average length of normal heart cycles during the measured period
NNmin	Shortest normal heart cycle during the measured period
PHY	Physical stress test
pNN50	Ratio of consecutive NN interval pairs with a greater difference than 50 ms
PSY	Psychological stress test
PTSD	Post-traumatic stress disorder
rMSSD	Root mean square of successive differences
SDNN	Standard deviation of normal heart cycles during the measured period
TP	Total power
VLF	Very-low-frequency band
VO_2_peak	Peak oxygen uptake
